# Efficacy of care manager-led support for family caregivers of people with dementia during the COVID-19 pandemic: a randomized controlled study

**DOI:** 10.1186/s12877-022-03371-2

**Published:** 2022-08-15

**Authors:** Kana Kazawa, Tatsuhiko Kubo, Hiroki Ohge, Shinya Ishii

**Affiliations:** 1 Department of Medicine for Integrated Approach to Social Inclusion, Graduate School of Biomedical and Health Sciences, Hiroshima University, Kasumi 1-2-3 Minami-ku, Hiroshima, 734-8553 Japan; 2 Department of Public Health and Health Policy, Graduate School of Biomedical and Health Sciences, Hiroshima University, Hiroshima, Japan; 3 Department of Infectious Diseases, Hiroshima University Hospital, Hiroshima, Japan

**Keywords:** Dementia, Family, COVID-19, Infection control measure, Preparedness, Care burden

## Abstract

**Background:**

A prolonged COVID-19 pandemic could exacerbate the risk of infection and undesirable effects associated with infection control measures of older people with dementia (PWD), and the care burden of families. In this study, we examined the efficacy of care manager-led information provision and practical support for families of older PWD who need care, regarding appropriate infection prevention, prevention of deterioration of cognitive and physical functions, and preparedness in cases of infection spread or infection during the pandemic.

**Methods:**

Fifty-three family members (aged ≥20 years) who were primary caregivers living with older PWD using public long-term care services were enrolled in an one-month randomized controlled trial. This duration was set based on behavior modification theory and with consideration of ethical issue that the most vulnerable people not benefiting from the intervention. The intervention group (IG) received care manager-led information provision and practical support, and the control group (CG) received usual care. Care burden (primary outcome) was measured using the Zarit Caregiver Burden Interview, and secondary outcomes were analyzed using Patient Health Questionnaire-9 (PHQ9), the Fear of COVID-19 Scale, and salivary α-amylase activity. Data were collected at baseline and after 1 month. Multiple regression analysis was conducted to examine the efficacy of the intervention. The participants evaluated the care managers’ support.

**Results:**

The participants were randomly divided into IG (*n* = 27) and CG (*n* = 26) groups. After the intervention, compared with the CG, there was a decrease in PHQ-9 (β = −.202, *p* = 0.044) and α-amylase activity in saliva (β = −.265, *p* = 0.050) in IG. IG also showed an increased fear of COVID-19 after the intervention (β = .261, *p* = 0.003). With the care managers’ support, 57.2% of the participants felt secure in their daily lives and 53.1% agreed that they were able to practice infection prevention suitable for older PWD.

**Conclusions:**

Our findings suggest that the care manager-led intervention may be useful for families of older PWD to enhance behavioral changes in preventing COVID-19 infection and improve their psychological outcomes in the COVID-19 era.

**Trial registration:**

This study was registered on April 2, 2021 (No. UMIN000043820).

**Supplementary Information:**

The online version contains supplementary material available at 10.1186/s12877-022-03371-2.

## Background

Older people living in the community, especially those with dementia, are the most vulnerable to the impact of measures such as social distancing and home isolation, which are aimed at preventing the spread of COVID-19 [1–3]. Shutting down or reducing paid services and changes in the environment for infection control measures have led to worsening cognitive function and behavioral and psychological symptoms of dementia (BPSD) in older people with dementia (PWD) [4–6]. Likewise, families of older PWD have reported feeling an increased physical and mental burden of caring, with anxiety about the infection risk of older PWD and themselves, and their daily care [5, 7]. Furthermore, in unprecedented circumstances, COVID-19-related information overload leads to stress and anxiety among families [8, 9]. Individualized and appropriate information and other support for older PWD and their families is very important.

In Japan, a super-aging society, the long-term care insurance system has been introduced in 2000 as a social security system to publicly provide services to support the increasing number of older people who need long-term care [10], and provides in-home care, day services, short stay, and services at long-term care facilities. However, during the COVID-19 pandemic, some long-term care facilities temporarily reduced or suspended services, mainly day services, to prevent the spread of infection and cluster outbreaks [11]. Older PWD, who are vulnerable due to cognitive decline or physical disabilities, often need support and medical care in their daily lives [12]. During the COVID-19, they need support for prevention of infection, prevention and mitigation of unfavorable effects that accompany infection control measures, and preparedness for emergencies such as the spread of infection or contracting infection. In the Japanese long-term care insurance system, care managers, who are in charge of care planning and care coordination for the persons certified for long-term care insurance [13], are the most suitable to provide this support. One care manager is assigned to each person certified for long-term care insurance, and most of the payments to them are from long-term care fees. The long-term care insurance act prescribes that care managers receive regular education about dementia, dementia care, the social security system, including long-term care insurance and community resources. They can provide person-centered information, including basic infection control measures based on the individual’s condition, and care coordination for them and their families.

It has been reported that information provision on infection prevention and physical activities at home [14], intervention using games to stimulate cognitive functions, and social interaction support using videocalls for older PWD helped in improving their physical and psychological outcomes [15–17]. However, there are no reports that include information and practical support on how to prepare for cases in which the infection spreads, care services are reduced, or older PWD and their families are infected.

Therefore, this study aimed to examine the efficacy of care manager-led information provision and practical support for families of older PWD who need care, regarding appropriate infection prevention according to the symptoms and activities of daily living of PWD, prevention of deterioration of cognitive and physical functions at home, and preparedness in case of infection spread or infection.

## Methods

### Study design

A randomized controlled trial was conducted between April and December 2021 in Hiroshima, Japan. Randomization was conducted using Electronic Data Capture (REDcap) by a researcher not associated with the study to ensure quality and reduce researcher bias. Care managers are often responsible for more than one older PWD (people who are cared for by the participants). Therefore, care managers were randomized into the intervention group (IG) or control group (CG), together with the families of older PWD they were responsible for.

The study protocol was approved by the Ethics Committee of Hiroshima University and registered on April 2, 2021 (ID: UMIN000043820). All procedures were performed in accordance with the approved protocol and the Declaration of Helsinki, and the Ethical Guidelines on Clinical Studies of the Ministry of Health, Labour, and Welfare of Japan.

### Participants

Participants were family members living with older PWD in Japan, who used public long-term care services, primary caregivers, and aged 20 years or older.

In this study, older PWD were defined as those who were aged 65 years and over, had been diagnosed with dementia, and had daily life independence level of Grade II or higher (conditions that need some kind of care, such as monitoring or direct care due to dementia symptoms) of older PWD under the long-term care insurance system. Participants were recruited in collaboration with care managers. Participants who did not live with older PWD or who had been diagnosed with dementia were excluded.

All participants provided written informed consent. While collecting information on attributes of older PWD, participants were asked to explain to the older PWD, after which the consent of older PWD or the substitute consent by the participant was obtained.

### Procedure

In this study, the durations of intervention and observation were set 1 month. We aimed to enhance the participants’ readiness for COVID-19-related self-management and to promote behavioral modification through the intervention. In Prochaska’s transtheoretical model, supporter discusses the factors that inhibit behavior change with the targeted person and provides them with personalized information to enhance his/her readiness to change their behavior and encourage action within 1 month [18]. We also considered the ethical issues that the most vulnerable people, older PWD, would not benefit from the intervention, and planned to provide support to CG after the observation period.

After consenting to participate in the study, participants completed a baseline questionnaire. For the IG, the care managers first provided COVID-19 related information on self-management (prevention of infection, prevention of deterioration of cognitive and physical functions, and preparedness in case of spread or infection) using a booklet developed by the researchers for the participants [19] (older PWD were also present, if possible), and discussed personalized practical plans with them in face-to-face meeting for about 30 minutes at the participants’ home. To prepare for the spread of the infection, or in case they or older PWD were infected, the participants were advised to compile information regarding older PWD’s conditions and care on self-completion sheets (Additional file 1) and share the information with other family members who were not usually involved in caregiving. Through these interventions, the care managers reassessed the conditions and environments of PWD comprehensively and coordinated their long-term care insurance services as needed. Besides, they shared information about PWD with multiple professions to address their care needs. We believed that this approach enabled other family members to care for older PWD when needed. Our protocol planned to switch from face-to-face meeting to remote using information and communication technology, such as tablets or phone calls, when the spread of infection became particularly severe. However, the meeting method was not switched because the care managers preferred face-to-face to visually observe the conditions of older PWD. To reduce the risk of infection, both the care manager and the participant wore masks, maintained physical distance of one–two meters, and ventilated the room. Next, the care manager followed up via biweekly phone calls of 15 minutes to assess the conditions and practices of older PWD and participants. During this period, usual long-term care insurance services were provided before enrollment.

The CG continued to use usual long-term care insurance services. As mentioned above, the CG received care managers’ support after the observation period to obtain the expected benefits of the intervention.

### Measures

The primary outcome was set as the total of Zarit Caregiver Burden Interview (ZBI) comprising 22 questions [20]. Responses are given using a 5-point Likert scale and scores range from 0 to 88, with higher total scores indicating higher care burden. The secondary outcomes were set as the Patient Health Questionnaire-9 (PHQ-9) [21], the Fear of COVID-19 Scale [22], salivary amylase activity for stress assessment, and anxiety and behavioral changes as process indicators. PHQ-9 is comprised of 9 items using a 4-point Likert scale and scores range from 0 to 27, with higher total scores indicating higher levels of depression. The Fear of COVID-19 Scale is comprised of 7 items using a 5-point Likert scale and scores range from 7 to 35, with higher total scores indicating higher levels of fear. α-amylase activity in saliva was measured using a Salivary Amylase Monitor (NIPRO Corporation), which consisted of a testing-strip, a salivary transcription device and an optical analyzer [23]. The participant inserted the testing-strip under the tongue and waited 30 seconds to collect 28 μl of saliva. In a range of salivary amylase activity between 0 and 200KU/L, the calibration curve for this monitor was reported to obtain R^2^ coefficient = 0.988 and coefficient of variation = 10.2% [23]. These were collected at baseline and 1 month later. Regarding anxiety and behavioral changes, the participants were asked whether the care managers’ support could mitigate their anxiety and promote their appropriate infection prevention practices. When conducting a randomized controlled trial, integrating quantitative data and qualitative process data contributes to generate an important insight about relationship between the intervention and outcomes [24]. The previous studies reported that families of older PWD felt anxiety about PWD’s risk of infection and physical and cognitive decline, and daily care for them, which caused their care burden [5, 7]. Therefore, we considered that these psycho-behavioral changes in the participants supported the context in which the change in primary outcome occurred.

Self-administered questionnaires were mailed to the participants’ homes. α-amylase activity in saliva was collected at the participants’ homes while adhering to the infection prevention protocol.

### Statistical analysis

For baseline comparison, Student’s t-test and chi-square test were performed between the two groups. To measure the efficacy of the intervention, multiple regression analysis was conducted with the scores of the outcome variables after 1 month as the dependent variable, using the group as the independent variable. As the assumptions of multiple regression analysis, we confirmed multicollinearity (variance inflation factor), outliner (values not within 3 standard deviations above or below the mean), and residual independence (Durbin-Watson test), homogeneity of variance (the relationship between residuals and fitted values) and normality (Normal Q-Q plot assessing if residuals are normally distributed, the histogram of residuals and Kolmogorov-Smirnov test) [25]. Then, Neuropsychiatric symptoms of PWD have been identified as the most important factors affecting caregiver burden and depression [26–28]. Therefore, the baseline values of the outcome variables and caregivers’ distress regarding BPSD of older PWD (Neuropsychiatric Inventory Questionnaire: NPI-Q subscale [29]) were used as adjustment variables to influence the outcome variables after 1 month. A per protocol analysis was performed.

The SPSS software (ver. 27.0; manufactured by IBM) was used for analysis, and the significance level was set at 5%.

## Results

### Participants’ characteristics

**Fig. 1:**
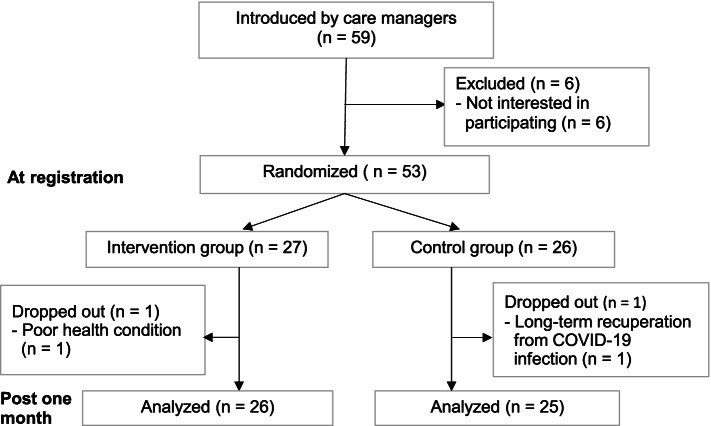
Flowchart of the study

Of the 59 participants recruited in collaboration with care managers, 53 who agreed to participate in the study were randomly assigned to the IG (*n* = 27) and CG (*n* = 26). One in the IG and one in the CG dropped out within 1 month after registration (Fig. 1).

Table 1 shows the results of the between-group comparison at baseline. There were no significant differences between any of the measures.

**Table 1:** Participants’ baseline characteristics

	Intervention group (*n* = 27)		Control group (*n* = 26)		*P*-Value
Participants					
Age, mean (SD)	67.3	(11.3)	65.7	(10.6)	0.737^a^
Gender, n (%)					
Male	8	(29.6)	6	(23.1)	0.757^b^
Female	18	(70.4)	20	(76.9)	
Relationship with person with dementia, n (%)					
Partner including spouse	12	(44.4)	10	(38.5)	0.905^b^
Child	13	(48.1)	14	(53.8)	
Child’s partner including spouse	2	(7.4)	2	(7.7)	
Older PWD cared by participants					
ADL: Barthel Index, mean (SD)	64.8	(23.4)	63.7	(11.3)	0.873^a^
The severity of dementia^a^, n (%)					
Moderate dementia	14	(51.9)	16	(61.5)	0.481^b^
Severe dementia	13	(48.1)	10	(38.5)	
NPI-Q total symptom score, mean (SD)	7.4	(4.6)	7.3	(7.2)	0.927^a^
NPI-Q distress score, mean (SD)	8.6	(6.7)	8.3	(9.9)	0.896^a^

*SD* standard deviation, *PWD* people with dementia, *ADL* activities of daily living, *NPI-Q* Neuropsychiatric Inventory Questionnaire

^†^The severity of dementia: It was classified as follows based on “Criteria for determination of the daily life independence level of the elderly with dementia “defined by Ministry of Health, Labour and Welfare (Japan). There are five levels in the original levels (Grade I to IV, and M)

- Moderate dementia (the original level Grade II); symptoms, behavior, or difficulty in communication that interfere with the person’s daily life are observed to some degree, but can live independently if someone will look after

- Severe dementia (Grade III or higher); symptoms, behavior, or difficulty in communication that interfere with the person’s daily life are observed once a while or frequently, and require care”

^a^Student t-test, ^b^chi-square test

### Efficacies of the intervention

Table 2 shows the changes in outcome measures. ZBI showed no change after 1 month, compared with the baseline in both groups. The mean [standard deviation (SD)] of PHQ-9 showed a decrease in the IG from 5.4 (5.0) at baseline to 4.0 (3.3) after 1 month, whereas the CG showed no change from 6.5 (5.8) at baseline to 6.8 (7.3) at 1 month. Regarding the Fear of COVID-19 Scale, the IG showed a slight increase from baseline 19.5 (5.1) to 20.5 (4.7) after 1 month, whereas the CG showed a decreasing trend from 18.1 (6.1) to 16.8 (5.5). In terms of α-amylase activity in saliva, there was no change between baseline and post one-month measures in the IG, but an increasing trend in the CG [from 2.6 (1.3) to 3.1 (1.3)].

**Table 2:** Changes in outcome measures one month after the intervention

Intervention group (*n* = 26), *CG*: control group (*n* = 25), mean (SD)							
		Baseline		Post one month		*P*-Value	
						Inter-group baseline comparison^a^	Within group pre- and post-comparison^b^
ZBI	IG	31.0	(14.7)	32.0	(13.4)	0.383	0.683
	CG	35.1	(18.6)	36.4	(19.2)		0.519
PHQ-9	IG	5.4	(5.0)	4.0	(3.3)	0.446	0.184
	CG	6.5	(5.8)	6.8	(7.3)		0.815
The Fear of COVID-19 Scale	IG	19.5	(5.1)	20.5	(4.7)	0.398	0.112
	CG	18.1	(6.1)	16.8	(5.5)		0.081
α-amylase activity in saliva^c^	IG	2.4	(1.1)	2.3	(1.3)	0.581	0.691
	CG	2.6	(1.3)	3.1	(1.3)		0.092

*SD* standard deviation, *IG* the intervention group, *CG* the control group, *ZBI* Zarit Caregiver Burden Interview, *PHQ-9* the Patient Health Questionnaire-9

^a^Student t-test

^b^paired t-test

^c^The raw values (KU/L) were converted to natural logarithms

To examine the efficacy of the intervention, multiple regression analyses were conducted for each of the primary and secondary outcome measures, with the score after 1 month as the dependent variable and the group as the independent variable, adjusted for the baseline score of the outcome measure and the NPI-Q distress score. The results of the regression analysis are shown in Table 3.

**Table 3:** Factors associated with outcome measures after one-month intervention in multiple regression analysis

	Coefficient (B)	95%CI	Standard coefficient (β)	*P*-Value	Adjusted R^2^
Primary outcome: ZBI					
**Group**^a^	**−1.427**	**(−7.452 ~ 4.597)**	**−.044**	**0.636**	0.587
Baseline outcome score	.701	(0.498 ~ 0.904)	.710	< 0.001^***^	
Baseline NPI-Q distress score	.257	(−0.145 **~** 0.659)	.130	0.205	
Secondary outcomes: PHQ-9					
**Group**^a^	**−2.302**	**(−4.543 ~ −0.061)**	**−.202**	0.044^*^	0.527
Baseline outcome score	.417	(0.194 ~ 0.640)	.393	< 0.001^***^	
Baseline NPI-Q distress score	.318	(0.175 **~** 0.462)	.464	< 0.001^***^	
Secondary outcomes: The Fear of COVID-19 Scale					
**Group**^a^	**2.782**	**(0.982 ~ 4.573)**	**.261**	0.003^**^	0.654
Baseline outcome score	0.715	(0.555 ~ 0.880)	.745	< 0.001^***^	
Baseline NPI-Q distress score	0.034	(−0.102 ~ 0.114)	.037	0.656	
Secondary outcomes: α-amylase activity in saliva^b^					
**Group**^a^	**−.704**	**(−1.409 ~ 0.000)**	**−.265**	**0.050**	0.192
Baseline outcome score	.447	(0.148 ~ 0.746)	.405	0.004^**^	
Baseline NPI-Q distress score	.016	(−0.026 ~ 0.059)	.103	0.445	

The values after one month of the outcome variables were used as the dependent variables

*CI* confidence interval, *ZBI* Zarit Caregiver Burden Interview, *NPI-Q* Neuropsychiatric Inventory Questionnaire, *PHQ-9* the Patient Health Questionnaire-9

^a^Using 1 for the intervention group and 0 for the control group

^b^The raw values (KU/L) were converted to natural logarithms

^*^p < 0.05

^**^p < 0.01

^***^p < 0.001

For the primary outcome, ZBI, the group was not significant (β = −.044, *p* = 0.636). The secondary outcome, PHQ-9, showed a significant decrease in the IG (β = −.202, *p* = 0.044); further, α-amylase activity in saliva also showed a decreasing trend (β = −.265, *p* = 0.050). In contrast, the Fear of COVID-19 Scale showed a slight but significant increase in the IG (β = .261, *p* = 0.003).

**Fig. 2:**
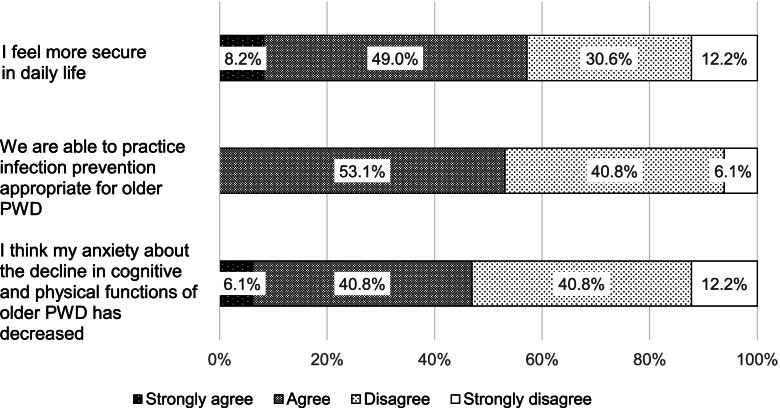
Participants’ evaluation of care managers’ support. *PWD*: people with dementia

In the questionnaire on care managers’ support (Fig. 2), 57.2% of the participants felt more secure in their daily lives. Moreover, 53.1% agreed that they were able to practice infection prevention suitable for older PWD, whereas 46.9% answered that their anxiety about the deterioration of cognitive and physical functions of older PWD had decreased.

## Discussion

Our findings showed that care manager-led information provision and practical support brought about a significant improvement in depressive tendencies, and a decreasing trend in stress (salivary amylase activity) among families of older PWD during the COVID-19 pandemic. There is no previous research on the experience of previous epidemics that includes support for the preparedness for shutting down or reduction in long-term care services due to the spread of infection, or if older PWD and their families become infected, in addition to prevention of infection and cognitive physical deterioration.

### Efficacies of care manager-led information provision and practice support for prevention and preparedness during the COVID-19 pandemic

In this study, the IG showed an improvement in the psychological outcomes. In the free response questionnaire, some participants answered that “they understood how to cope with anxiety” and “felt secure that they would be supported (by the professionals involved).” These results suggest that while the families were anxious and stressed due to an information overload about COVID-19, the care managers provided them with personalized and appropriate coping methods; further, they understood these methods, which may lead to an improvement in their depressive tendencies. Psychoeducational interventions aimed at understanding dementia and managing behavioral changes have been reported to improve family members’ knowledge of dementia care and cognitive-behavioral mood management [30], and this evidence may corroborate the efficacy of this study. In addition, families of older PWD play an important role in dementia care, not only in terms of financial and emotional support, but also in advocating for their rights [31, 32]. During the COVID-19 pandemic, families were required to make difficult decisions about care policies for the older PWD, while considering the risk of infection due to face to face contact with others and deterioration of the condition due to cancellation of paid service use [33, 34]. However, face-to-face contact with healthcare professionals was limited to the minimum necessary, and the fact that they were forced to change their communication methods had a psychological impact on the families’ anxiety and sense of isolation [35]. In such a situation, the support of the care managers based on the understanding of their conditions and lives may have reduced the families’ stress and conflicts, given them a sense of security, and improved their psychological outcomes.

Positive changes such as collaboration among informal and formal caregivers and strengthening their relationships may improve the quality of person-centered dementia care and lead to continuity of care [36]. We believe that this meaningful finding will be a useful strategy for supporting older PWD and their families during COVID-19 pandemic.

In spite of the short-term results, families were able to deepen their understanding of preventive practices and emergency preparedness, while enhancing their readiness to practice. Improving the chronic anxiety and stress of families can lead to a reduction in their risk for various physical and psychological morbidities, including cardiovascular diseases and depression [26]. Furthermore, it was reported that support for PWD and their families’ psychosocial and physical well-being at home (4 weeks) was beneficial for improving cognitive function of older PWD and their quality of life under COVID-19 [30]. We believe that changes in families’ practices through this intervention will contribute to older PWD’ health. In the long term, families may practice COVID-19-related personalized dementia care and reinforce practices due to perceived benefits, such as improving the behavioral, psychosocial and health outcomes of older PWD.

However, the IG showed a slight increase in fear of COVID-19 after the intervention. This may be due to differences in the spread of infection in these regions. In this study, we did not evaluate the number of positive cases or other indicators regarding the severity of the infection spread; therefore, future studies should include such regional factors.

### Implications for proactive support to protect the lives of older PWD and their families

Under the COVID-19 pandemic, the supply of formal services such as long-term care insurance was reduced [11], and connections among local residents became weaker. As a result, the content and delivery system of care for older PWD had to be changed, and the health risks for them and their families increased [37, 38]. For older PWD and their families who need care, changes in their familiar care contents and environment increased their anxiety and stress [39]. In this study, care managers and the families of older PWD held discussions about readjustment of care, including long-term care insurance services, and emergency preparedness in case of limited access to services. For community-dwelling older PWD who need care and their families, knowing the potential risks and disruptions in the event of a disaster and taking preventive measures could help reduce its impact [40, 41]. This proactive and preventive practice will contribute to their sense of control over their own health and lives in a prolonged and unpredictable pandemic. We also believe that the findings of this study suggest that this support may be useful not only in the COVID-19 pandemic, but also in other disasters.

### Limitations

There were some limitations to this study. First, the characteristic neuropsychiatric symptoms associated with the primary disease of PWD are related to caregiver burden [26], but the primary disease was not assessed in this study. Second, we were not able to observe behavioral changes based on this intervention because we thought that non-interventions would be disadvantageous for the CG and the evaluation was set in a short period of 1 month. Third, the results may not be generalized because the sample size is small. During the study period, the spread of COVID-19 became serious in the target field, and the care managers focused on maintaining the usual provision of long-term care insurance services. This made recruitment difficult.

Therefore, future studies should ensure a sufficient sample size and examine the long-term effects of the interventions. Validation of the effects after stratification by primary disease may provide implications for further improvements in individualized dementia care.

## Conclusions

Care manager-led information provision and practical support during the COVID-19 pandemic showed a significant improvement in depressive tendencies and a decreasing trend in stress among families of older PWD. Many participants reported that they felt security in their daily lives and were able to practice infection prevention suitable for older PWD through the intervention.

COVID-19-related personalized dementia care may improve the health outcomes of older PWD and their families. Future studies could evaluate the effect of care manager-led information provision and practical support on the behavioral changes, and assess if the long-term effects of interventions would sustain after the COVID-19 era.

## Supplementary Information

**Additional file 1.**

## Data Availability

The datasets generated and/or analyzed during the current study are not publicly available because no consent for secondary use of the data was obtained from the participants. However, the datasets are available from the corresponding author on reasonable request.

## Abbreviations

BPSD: Behavioral and psychological symptoms of dementia
PWD: People with dementia
IG: The intervention group
CG: The control group
ZBI: Zarit Caregiver Burden Interview
PHQ-9: The Patient Health Questionnaire-9
NPI-Q: Neuropsychiatric Inventory Questionnaire
SD: Standard deviation
