# Supplementary material for: Efficacy of care manager-led support for family caregivers of people with dementia during the COVID-19 pandemic: a randomized controlled study
Source: BMC Geriatr. 2022 Aug 15;22:671. doi: 10.1186/s12877-022-03371-2 (PMC9376895; doi:10.1186/s12877-022-03371-2)
Supplement: Supplementary file 1 — Additional file 1. [file 12877_2022_3371_MOESM1_ESM.pdf]

Our preparedness during the epidemic

-Preparedness for shut down or reduction in long-term care services due to the spread of infection, or in case you and your family are infected.

1． Let’s write down the kinds and frequencies of long-term care services that your family with dementia uses.

2． Let’s recheck the purpose of using each service.

For example,

- To receive care for daily living such as meals, bathing, and elimination.
- To manage illnesses and wounds such as medication, injections, treatment of bedsores, etc.
- To check on physical condition.
- For rehabilitation.

3． Let’s discuss the priorities of the services your family uses, and check the services that should not be interrupted (high priority services).

4． Let’s consider alternatives in case of a shut down or reduction in long-term care services

- Check the local long-term care providers that can respond to your family's condition (severity of dementia, medical and long-term care that your family needs, etc.).
- Discuss who will be involved in care if a responsive care provider cannot be found.

Next, let us consider the support that your family needs. It is also useful to write down what kind of care you or your family would like to receive in case of infection.

5. Let us write down your family's life rhythms and routine.

- Changes in your family's life rhythm and routines can lead to stress, anxiety, sleep disturbances, as well as worsening of dementia symptoms and physical conditions.

✓ **Are there different life rhythm patterns? (e.g., with or without day services).**
✓ **Are there any routines that are particularly important to your family?**

Case. The day without day services

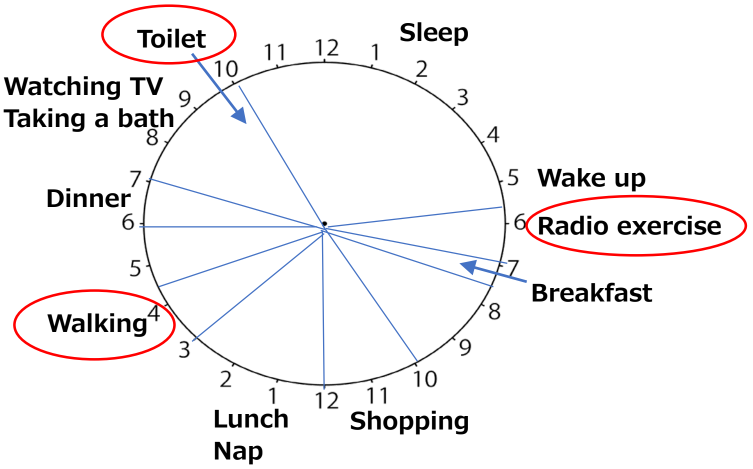

Pattern 1
( )

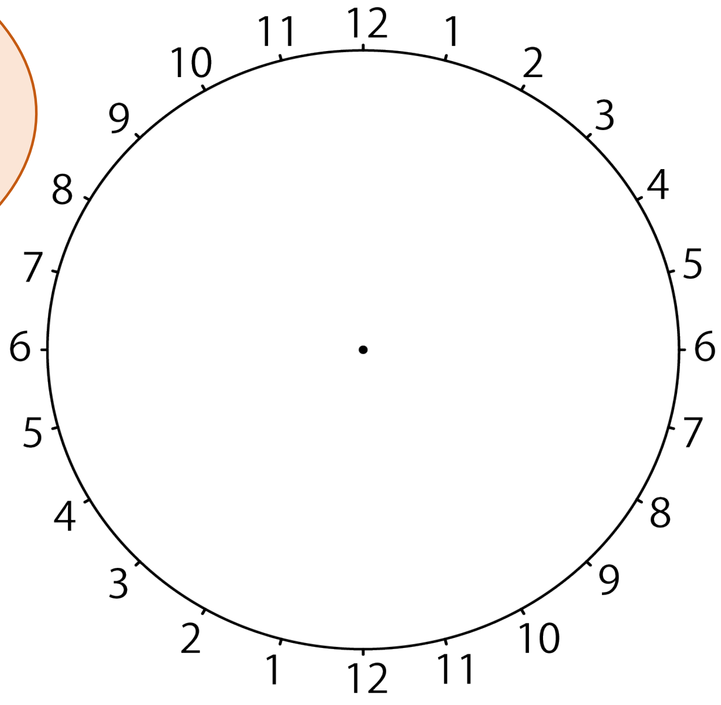

Pattern 2
( )

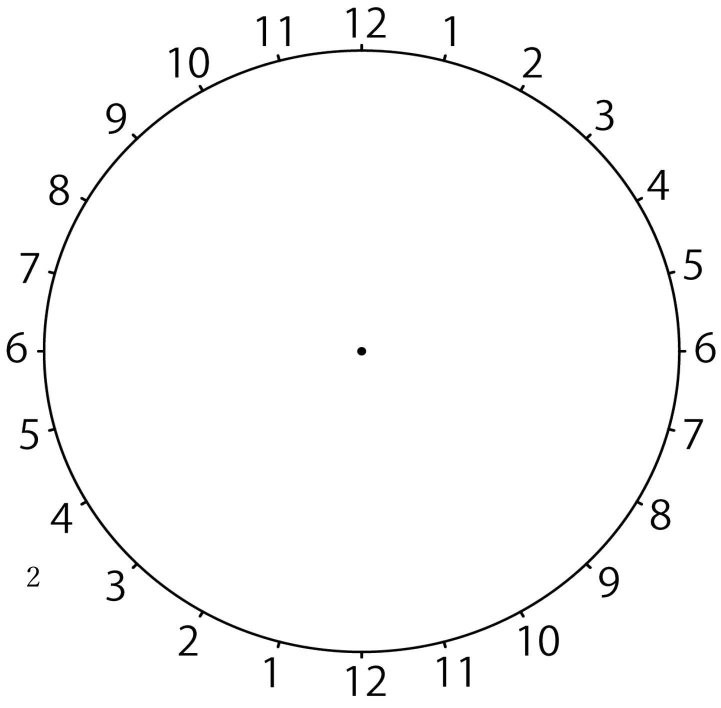

Disease management

- **Hospitals/clinics where your family receives regular medical care:**

- **Your family's attending doctor:**

Let's check if home-visit medical care is available when needed.

- **Medication and injection:**

◆ **Medication:**

(Upon waking Before/after breakfast Before/after lunch
Before/after dinner Between meals Others ( ))

◆ **Injection:**

◆ **Points to note medication and injections**

For example,
- If the person has difficulty swallowing medication, wrap the medication in a jelly or ointment, and take it.
- Mix the medication with food if the person refuses to take it*.
- What medications can and cannot be skipped when the person is unable to take them and what to do about it?

If a person with diabetes uses oral medication or insulin to control blood glucose,
- If the person does not eat or eat very little, be aware of hypoglycemia symptoms, and consult the doctor as soon as possible about the amount of medication or injections he/she should take.

*As the combination of medication and food may make the taste bitter or affect the effect of the medication, check with your doctor or pharmacist and do not make judgements based on your own assumptions. In addition, some medicines should not be crushed because they are difficult to swallow.

Long-term care

- **Write down where you will buy the care equipment you need (diapers, etc.), where you will store it in your house, and how you will use it.**

- **If your family uses long-term care insurance services, let's check with the service provider for points of care.**

  For example
  - Meals (how to prepare them, how to provide assistance and oral care)
  - Defecation (assistance with transfers, encouragement to go to the toilet, enemas, and stool removal)
  - Environment (temperature and humidity of the room, ventilation), etc.

- **If several caregivers are involved in caregiving, let's discuss how and when each will provide support (support for daily living or disease management).**
  Let us also discuss the case in which the primary caregiver cannot be involved in caregiving of your family with dementia due to infection or other reasons.

＊Preparedness in case your family is infected.

If your family with dementia is infected, other families may be very upset.

It is also important to write down your family's and other families' thoughts, values, and what kind of treatment your family would like to receive and share them in advance with your loved ones and the medical care professionals involved.
